# Liniment for Fissure of the Anus

**Published:** 1872-01

**Authors:** 


Liniment for Fissure of the Anus.—M. Van Holsbeck (Medical News) states that he has succeeded in curing anal fissures with the following application, which had resisted the division of the sphincter: Dissolve one part of tannic acid in sixteen parts of glycerine. A tent wet with this preparation is to be introduced into the rectum night and morning. The bowels are to be kept open.

